# Synthesis of Anti-Inflammatory Drugs’ Chalcone Derivatives and a Study of Their Conformational Properties Through a Combination of Nuclear Magnetic Resonance Spectroscopy and Molecular Modeling

**DOI:** 10.3390/ph18010088

**Published:** 2025-01-13

**Authors:** Nikitas Georgiou, Andromachi Tzani, Kyriaki Vavougyiou, Christos Papadopoulos, Nikolaos Eleftheriadis, Primož Šket, Demeter Tzeli, Tuomas Niemi-Aro, Anastasia Detsi, Thomas Mavromoustakos

**Affiliations:** 1 Laboratory of Organic Chemistry, Department of Chemistry, National and Kapodistrian University of Athens, Panepistimioupolis Zografou, 11571 Athens, Greece; nikitage@chem.uoa.gr (N.G.); k.vavougyiou@outlook.com (K.V.); 2 Laboratory of Organic Chemistry, Department of Chemical Sciences, School of Chemical Engineering, National Technical University of Athens, Heroon Polytechniou 9, Zografou Campus, 15780 Athens, Greece; atzani@mail.ntua.gr (A.T.); adetsi@chemeng.ntua.gr (A.D.); 3 Department of Chemistry, University of Crete, Voutes, 70013 Heraklion, Greece; chemp1181@edu.chemistry.uoc.gr (C.P.); n.eleftheriadis@uoc.gr (N.E.); 4 Slovenian NMR Centre, National Institute of Chemistry, SI-1001 Ljubljana, Slovenia; primoz.sket@ki.si; 5 Laboratory of Physical Chemistry, Department of Chemistry, National and Kapodistrian University of Athens, Panepistimioupolis Zografou, 11571 Athens, Greece; tzeli@chem.uoa.gr; 6 Theoretical and Physical Chemistry Institute, National Hellenic Research Foundation, 48 Vassileos Constantinou Ave., 11635 Athens, Greece; 7 Institute of Biotechnology, Helsinki Institute of Life Sciences, Viikinkaari 1, P.O. Box 65, University of Helsinki, 00014 Helsinki, Finland; tuomas.niemi-aro@helsinki.fi

**Keywords:** chalcones, inflammation, LOX, NMR, molecular dynamics

## Abstract

Background: In this study, two chalcone analogs were synthesized through in silico and experimental methods, and their potential to inhibit the lipoxygenase enzyme, which plays a role in the inflammation pathway, was assessed. Specifically, this study is a continuation of previous research in which chalcone derivatives were synthesized and characterized. Objectives/Methods: In the current work, we present the re-synthesis of two chalcones, with a focus on their docking studies, NMR analysis, and dynamic simulations. The structure of each chalcone was elucidated through a combination of Nuclear Magnetic Resonance (NMR) and Density Functional Theory (DFT). The substituent effect on the absorption spectrum of the two chalcone derivatives was studied. Results: A “LOX–chalcone” complex, predicted by docking studies, was further examined using molecular dynamics (MD) simulations to evaluate the stability of the complex. After fully characterizing the “LOX–chalcone” complexes in silico, the atomic details of each chalcone’s interaction with LOX-1 and 5-LOX were revealed through Saturation Transfer Difference (STD) NMR (Nuclear Magnetic Resonance). Finally, their selectivity profile was investigated against human 15-LOX-1 and general Lipoxidase activity. Conclusions: The in silico methods suggest that chalcones could be promising lead compounds for drug designs targeting the LOX enzyme.

## 1. Introduction

**Scheme 1:**
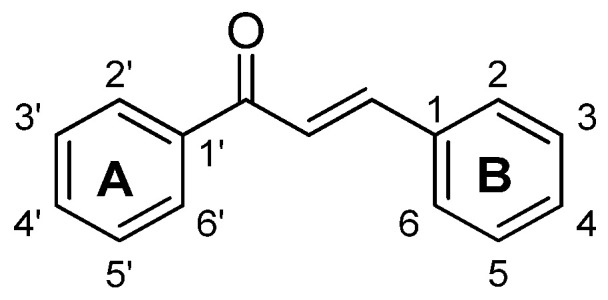
General structure and numbering scheme of chalcones.

Chalcones are a large and substantial group of bioactive compounds that are among the leading categories of the flavonoid family. They consist of two aromatic rings (A and B) connected by a α,β-unsaturated carbonyl system (Scheme 1). Chalcones possess a broad spectrum of biological activities, such as antioxidant, antidiabetic, anti-inflammatory, neuroprotective, antiviral, antimalarial, antimicrobial, and antifungal activities [1,2,3,4,5,6].

Chalcones have been extensively used as lead compounds for the discovery of new drugs in medicinal chemistry, and studies have shown that the free hydroxyl groups present in aromatic rings A and B are essential to increase their biological activity [4].

2’-hydroxy-chalcones are characterized by a hydroxyl group (-OH) attached to the second carbon atom of the A ring. The presence of the hydroxyl group can influence the chemical properties and biological activities of these compounds [7]. 2’-hydroxy-chalcones have been studied, focusing on their various biological activities, such as their anti-inflammatory [6,8] anticancer [9], and antioxidant [3,4,7] properties, as well as their potential use as inhibitors of lipoxygenases (LOXs) [3,4]. Studies of compounds with LOX inhibition activity highlight their potential as valuable pharmacological agents.

LOX are iron-containing enzymes widely found in plants and animals. They catalyze the oxygenation of polyunsaturated fatty acids such as arachidonic acid (in mammals) and linoleic acid (in plants), generating lipid hydroperoxides. LOX inhibitors are of great interest due to their potential involvement in various pathophysiological conditions. Therefore, LOX constitutes a potential target for rational drug design and in the discovery of inhibitors for the treatment of variety of disorders. Moreover, Lipoxygenase (LOX) enzymes play a significant role in various physiological and pathological processes by catalyzing the oxidation of polyunsaturated fatty acids into bioactive lipid mediators. These mediators are implicated in inflammation, cancer progression, and cardiovascular diseases. Therefore, inhibiting LOX activity can have therapeutic implications, such as reducing inflammation, preventing tumor growth, or mitigating oxidative stress-related damage. Understanding LOX’s role in these pathways provides a basis for the development of targeted inhibitors as potential treatments for related disorders [3,10,11].

**Scheme 2:**
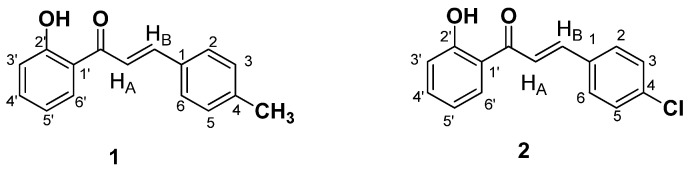
Structures of chalcones **1** and **2**.

In continuation of previous findings regarding the ability of various 2’-hydroxy-chalcones to act as soybean LOX inhibitors [3,4,12], in this work, two 2’-hydroxy-chalcones possessing a 4-methyl and a 4-chloro substituent in ring B (Scheme 2, chalcones **1** and **2**) were selected and re-synthesized with a modified optimized procedure for further study, implementing a combination of NMR spectroscopy and computational analysis.

These studies were conducted in an effort to identify the effect of different structural characteristics that possibly contribute to the inhibition of LOX-1, LOX-5, and LOX-15 activity. According to the literature, the activity of 2′-hydroxy-chalcones is enhanced by the introduction of substituents such as a chloro- or a methyl group at position 4 of ring B [13]. Thus, in an effort to study the effect of the presence of substitutions on aromatic ring B, in the present study, two chalcones with different substitutions in position 4 of ring B were synthesized. Specifically, chalcone **1** possesses an electron-donating methyl group in position 4 of ring B, while chalcone **2** bears an electron-withdrawing Cl group at the same position.

The interaction profile of the studied chalcones with LOXs was revealed through Saturation Transfer Difference (STD) NMR (Nuclear Magnetic Resonance) and molecular docking and molecular dynamics simulations. A conformational analysis was carried out using density functional theory (DFT) calculations. Finally, the in vitro activity of the synthesized chalcones at LOX-1 and LOX-15 was evaluated.

These studies are important for determining the compounds’ ability to bind and remain stable within the enzyme’s active site. Additionally, the findings of the present work can provide a more comprehensive understanding when designing new chalcones with optimized LOX-inhibition activity.

## 2. Results

### 2.1. Structure Elucidation of Chalcones **1** and **2**

The identification of chalcone **1** started with the two doublets at 7.83 and 8.05 ppm, which correspond to H_A_ and H_B_, as previously reported in the literature [3]. Through 2D-NOESY (Figures S1 and S2), H2 and H3 were identified, and H_B_ was shown to correlate with H2. Through 2D-HSQC (Figures S3 and S4), the corresponding carbons are also identified, because they exhibited ^1^J_C-H_ coupling with the corresponding protons. A correlation was then observed in 2D-NOESY between H_A_ and a proton at 8.25 ppm. This corresponds to H6′, because it is the only proton which is in close vicinal proximity to H_A_ and presents a doublet because it correlates to one proton. Through 2D-NOESY from H6’, the remaining protons were identified. Through 2D-HSQC, the corresponding carbons were also identified, because they showed ^1^J_C-H_ coupling with the corresponding protons. From the two-dimensional spectrum ^13^C-^1^H 2D HSQC of the studied molecule, all the carbons in the molecule were identified except for the quaternary and carbonyl carbons. The remaining carbons could be identified via ^13^C-^1^H 2D HMBC (Figure S5). It was observed that H_A_ showed a ^2^J_C-H_ correlation with C1, H2 showed a ^3^J_C-H_ correlation with C4, and H6′ showed a ^3^J_C-H_ correlation with C2′ and a ^2^J_C-H_ correlation with C1′. With this approach, a comprehensive assessment of every proton and carbon atom of chalcone **1** was successfully carried out.

**Figure 1:**
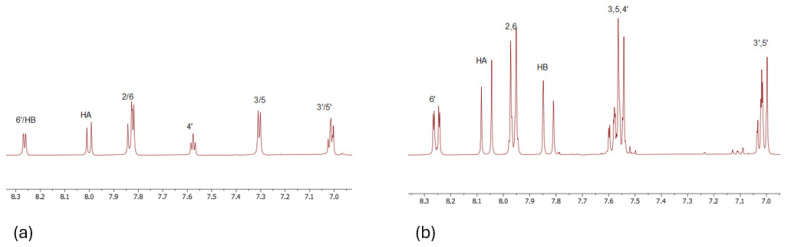
^1^H NMR spectra in DMSO of (**a**) **1** and (**b**) **2**. The spectra were recorded in DMSO-d_6_ on a Bruker AC 850 MHz spectrometer at 25 °C.

A similar procedure was carried out for chalcone **2** (Figures S6–S9). All the calculations were performed using DMSO-d_6_. The two proton spectra of each compound are shown in Figure 1. Moreover, Table 1 shows the chemical shifts in the two compounds.

### 2.2. Results of Conformational Analysis

DFT was employed to investigate the lowest-energy conformations of chalcones **1** and **2** due to its accuracy compared to the other available methods. Multiple initial structures underwent geometry optimization, yielding a total of eight conformers for each compound. After the lowest-energy conformational analysis, the distances between the protons that exhibited correlation signals in space in the 2D-NOESY were calculated. These distances indicated that the specific protons are indeed in spatial proximity, justifying the correlations observed in the NOE spectra. Based on the calculated energy levels, the molecular structures, and the correlations observed in the 2D-NOESY spectra, the lowest-energy conformations depicted in (a) and (b) are considered to most likely represent chalcones **1** and **2**, respectively.

**Figure 2:**
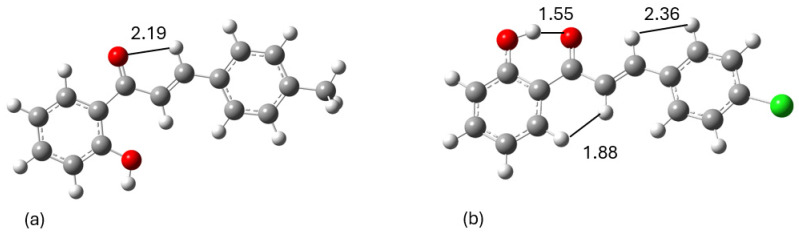
The lowest-energy arrangements for (**a**) **1** and (**b**) **2**, along with their spatial correlations, which dictate their lowest-energy configurations; distances in Å for the DFT (BP86/def2-SVP) methodology (red is oxygen, green is chlorium).

According to the results, the *trans* conformation is the one that agrees most with the experimental results. In the comparative analysis of the two molecular conformations (Figure 2), it is observed that the orientation of the carbonyl group relative to the phenolic hydroxyl group differs between the structures. In the lowest-energy conformation of chalcone **1**, the carbonyl group is oriented so that its oxygen atom points away from the phenolic hydroxyl group, resulting in an anti-periplanar arrangement. Conversely, in the lowest-energy conformation of chalcone **2**, the carbonyl group is oriented in the same direction, with its oxygen atom positioned toward the phenolic hydroxyl group, forming a hydrogen bond. In Table 2, the proton correlations observed via 2D-NOESY for the two compounds are described, along with their respective distances.

### 2.3. Docking Calculations

Using the Glide/XP algorithm for induced fit docking (IFD), numerous conformations were generated for the docking of these two molecules in LOX-1, 15-LOX, and 5-LOX, sorted using the decreasing IFD Score scoring function (Table 3). This scoring function considers not only the strength of the ligand’s binding to the protein’s cavity but also the prime energy of the protein in all protein–ligand complexes.

Conformers with the lowest energy were selected as the optimal docking pose for subsequent MD simulations. This conformation was preferred because it shows the direct interaction of the ligand with the active site, and its low IFD score indicates that the protein–ligand complex is the most stable. Additionally, both energetically and structurally, this cluster of conformers shows no significant differences in their binding modes.

**Figure 3:**
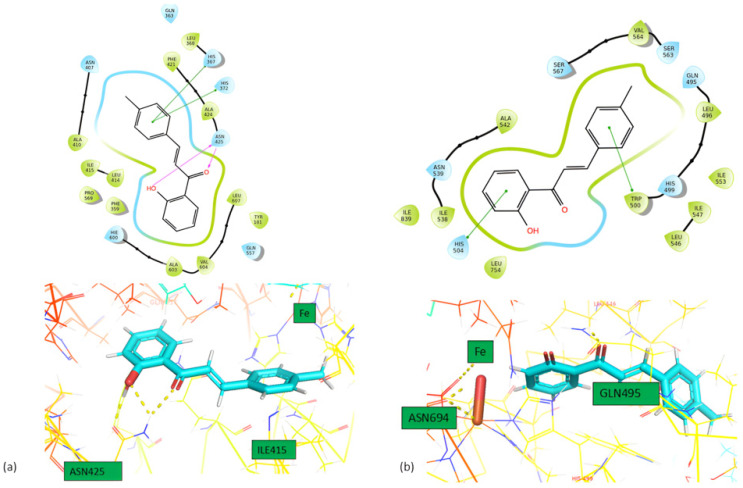
Interactions of **1** with (**a**) 5-LOX and (**b**) 1-LOX in 2D (**above**) and 3D (**bottom**) formats.

**Figure 4:**
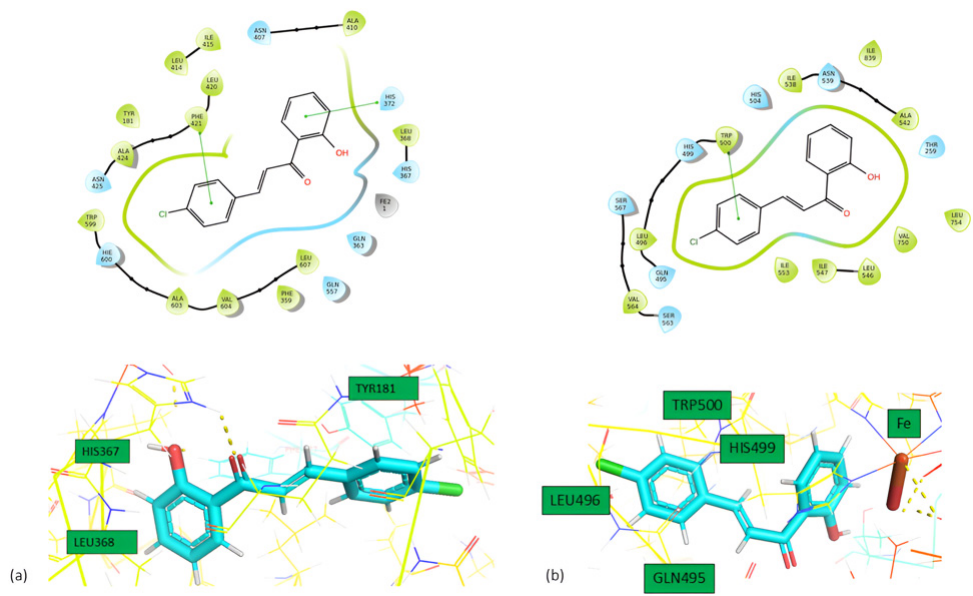
Interactions of **2** with (**a**) 5-LOX and (**b**) 1-LOX in 2D (**above**) and 3D (**bottom**) formats.

**Figure 5:**
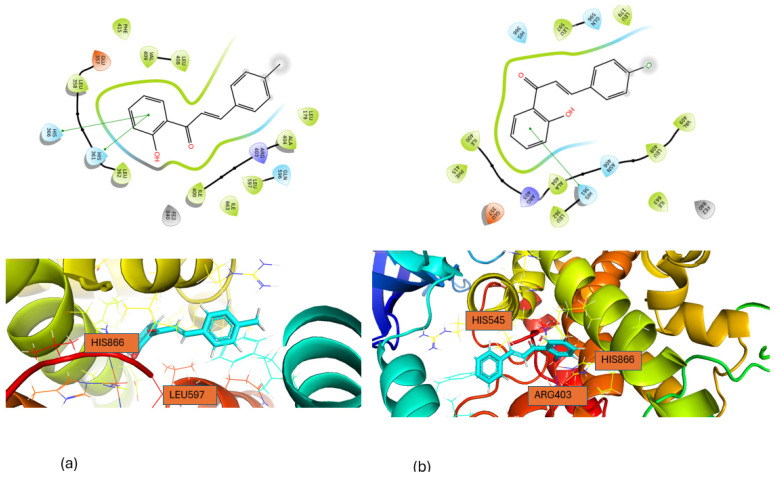
Interactions of **1** with (**a**) 15-LOX and **2** with (**b**) 15-LOX in 2D (**above**) and 3D (**bottom**) formats.

From the calculated binding energies, all the molecules are predicted to bind strongly to the active center of 5-LOX and LOX-1. Specifically, **1** seems to form three hydrogen bonds between the hydroxyl and carbonyl group and ASN425 amino acid in LOX-5, and also two π-π stacking configurations with HIS367 and HIS372 amino acids (Figure 3). In LOX-1, **1** forms two π-π stacking interactions with HIS504 and TRP500 amino acids. Finally, **2** likely forms two hydrogen bonds between the carbonyl group and HIS367 amino acids and two π-π interactions with HIS372 and PHE421 amino acids (Figure 4). Moreover, in LOX-1, it has one π-π interaction with the TRP500 amino acid. However, both compounds do not bind strongly to the active center of 15-LOX. Specifically, compound **1** is suggested to form two pi-pi interactions with HIS361 and HIS366 and compound to form **2** one pi-pi interaction with HIS361 amino acid (Figure 5). This is the reason we did not proceed with the STD experiments on 15-LOX. All the interactions between these two molecules and the enzymes are shown in Table 4. Our study reveals that the studied chalcones exhibit a significantly stronger inhibition of LOX-1 compared to other previously studied chalcones. This superior inhibitory activity suggests that our chalcone has a more effective interaction with the LOX-1 binding site, likely due to its unique structural features. As a result, it may offer greater potential as a therapeutic agent targeting LOX-1-related pathways [14].

### 2.4. Results of Molecular Dynamics

Molecular dynamics simulations were carried out to assess the stability of the compounds’ orientation, as suggested by the docking studies. The results of the MD simulation for the protein–ligand complex demonstrated the stable binding of all the compounds within the cavity of LOX. To quantify this finding, the Root Mean Square Deviation (RMSD) of each ligand was calculated with reference to its initial docking position. Over the course of the 200 ns simulation, each ligand remained bound to the binding site of both enzymes. The sustained binding of each compound within the cavity throughout the simulation confirms our docking predictions, indicating that they could serve as inhibitors to LOX and hold promise as potential LOX inhibitors.

**Figure 6:**
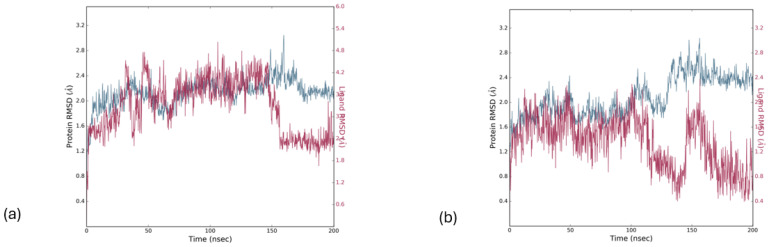
RMSD values for the protein (5-LOX) (depicted in green) and ligands 1 (**a**) and 2 (**b**) (depicted in magenta) were calculated over a 200 ns simulation period, with the initial docking pose serving as the reference structure (upper image).

In Figure 6, the protein’s Ca atoms demonstrate a low RMSD value (<4.0 Å), indicating favorable convergence of the system, while the RMSD of the ligand is also depicted for the 200 ns duration of MD simulation. The initial docking pose served as the reference when measuring the RMSD of the ligand’s heavy atoms. Throughout the entire simulation period, the ligand exhibited remarkable stability, with an RMSD value of approximately (<4.0 Å), as illustrated in Figure 6. Moreover, from the timeline graph (Figure S10), it was found that both compounds maintained a relatively stable number of contacts throughout the simulation, with occasional fluctuations, suggesting dynamic interactions within the binding pocket. In compound **1**, key residues such as TYR181, LEU420, and PHE421 showed persistent and strong interactions, suggesting they play a crucial role in stabilizing the ligand. In contrast, residues like GLN363, LEU414, and ASN425 exhibited more intermittent contacts, which may reflect transient interactions or structural flexibility. In compound **2**, the heatmap showed variability in its new contacts, with some residues (e.g., HIS372 and HIS603) maintaining consistent interactions, while others (e.g., VAL604 and ILE673) exhibited transient, high-intensity contact.

**Figure 7:**
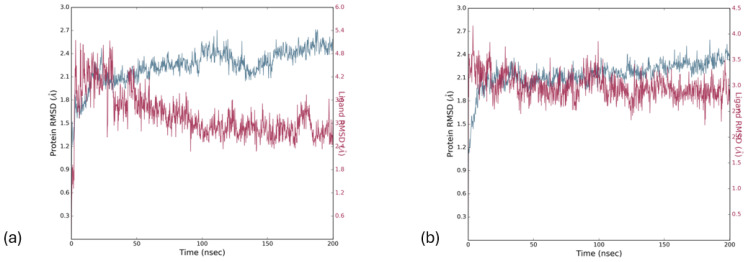
RMSD values for the protein (LOX-1) (depicted in green) and ligands 1 (**a**) and 2 (**b**) (depicted in magenta) were calculated over a 200 ns simulation period, with the initial docking pose serving as the reference structure (upper image).

In Figure 7, the protein’s Ca atoms demonstrate a low RMSD value (<4.0 Å), indicating favorable convergence of the system, while the RMSD of the ligand is also depicted for the 200 ns duration of the MD simulation. The initial docking pose served as the reference when measuring the RMSD of the ligand’s heavy atoms. Throughout the entire simulation period, the ligand exhibited remarkable stability, with an RMSD value of approximately (<4.0 Å), as illustrated in Figure 7. Moreover, the timeline (Figure S11) for compound 1 shows a fluctuation in the number of starting contacts, ranging between 6 and 12, indicating a moderately dynamic interaction between the ligand and binding pocket. The consistent interactions between residues such as LEU496 and ILE553 are highlighted, suggesting their importance in stabilizing the ligand. Conversely, residues like ALA542 and ILE839 show more transient, sporadic contact, reflecting regions of flexibility or dynamic interaction. Compound 2 showed consistent interactions with residues ILE839, HIS690, and ASN694, while it had more sporadic interactions with ILE837 and ALA542.

**Figure 8:**
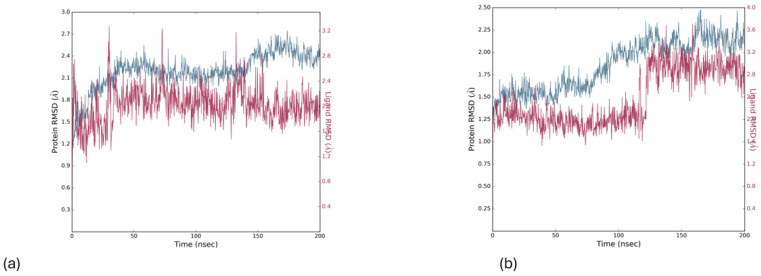
RMSD values for the protein (15-LOX) (depicted in green) and ligands 1 (**a**) and 2 (**b**) (depicted in magenta) were calculated over a 200 ns simulation period, with the initial docking pose serving as the reference structure (upper image).

In Figure 8, protein’s Ca atoms demonstrate a low RMSD value (<4.0 Å), indicating the favorable convergence of the system, while the RMSD of the ligand is also depicted for the 200 ns duration of the MD simulation. The initial docking pose served as the reference when measuring the RMSD of the ligand’s heavy atoms. Throughout the entire simulation period, the ligand exhibited remarkable stability, with an RMSD value of approximately (<4.0 Å), as illustrated in Figure 8. From the timeline (Figure S12), it seems that compound 1 had more consistent interactions with HIS541, ILE663, and HIS361 during the simulation period, while it had more sporadic interactions with ILE593 and GLN596. Compound 2 had more consistent interactions with HIS361, HIS541, and ILE663 and more sporadic interactions with ILE414 and GLN548.

### 2.5. Charting the Compound–LOX-1/5-LOX Interactions Through the Saturation Transfer Difference (STD) NMR Experiment

After establishing the effectiveness of these compounds in inhibiting LOX-1 and 5-LOX, our aim was to delve deeper into their direct interactions and precisely identify the epitope involved in the binding with soybean LOX-1 and 5-LOX. To investigate this interaction, we employed Saturation Transfer Difference (STD) NMR, a technique capable of assessing a ligand’s potential interaction with a pharmaceutical target and pinpointing the specific protons involved in the molecular binding process.

**Figure 9:**
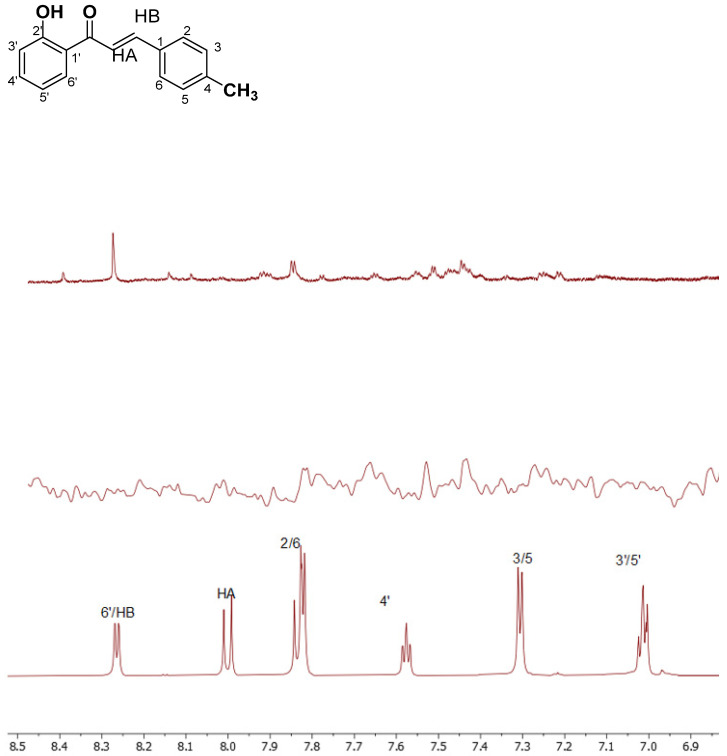
(Top) The STD-NMR reference spectrum of **1** (1000 mM) in the presence of 5-LOX protein (1 mM) at a 1:1000 ratio with respect to **1** was obtained in a potassium phosphate buffer at pH 7.2 and 600 μL D_2_O using an 850 MHz NMR spectrometer at 25 °C. (Middle) The STD-NMR reference spectrum of **1** (1000 mM) in the presence of the LOX-1 protein (1 mM) at a 1:100 ratio with respect to **1** was obtained in potassium phosphate buffer at pH 7.2 and 600 μL D_2_O using an 850 MHz NMR spectrometer at 25 °C. (Bottom) ^1^H spectra of **1** in DMSO, obtained using an 850 MHz NMR spectrometer at 25 °C.

**Figure 10:**
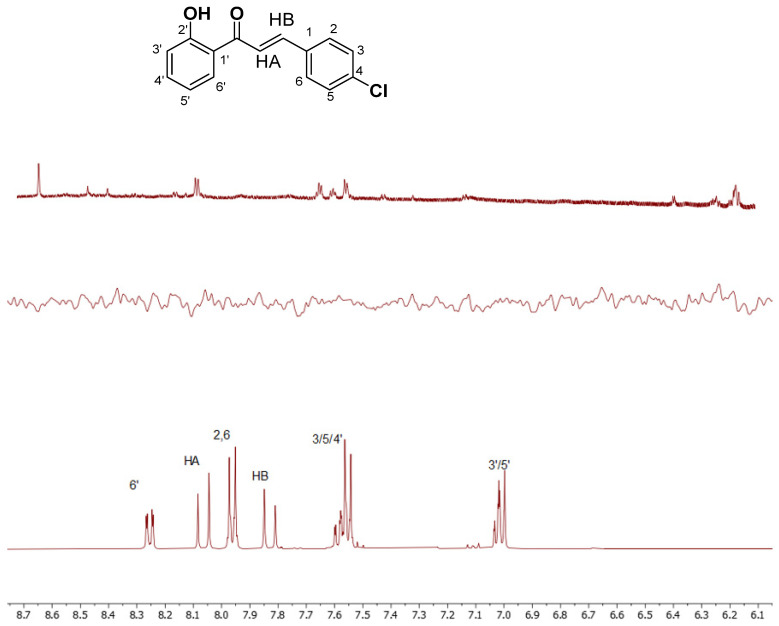
(Top) The STD-NMR reference spectrum of **2** (1000 mM) in the presence of 5-LOX protein (1 mM) at a 1:1000 ratio with respect to **2** was obtained in potassium phosphate buffer at pH 7.2 and 600 μL D_2_O using an 850 MHz NMR spectrometer at 25 °C. (Middle) The STD-NMR reference spectrum of **2** (1000 mM) in the presence of LOX-1 protein (1 mM) at a 1:100 ratio with respect to **2** was obtained in potassium phosphate buffer at pH 7.2 and 600 μL D_2_O using an 850 MHz NMR spectrometer at 25 °C. (Bottom) ^1^H spectra of **2** in DMSO, obtained using an 850 MHz NMR spectrometer at 25 °C.

The results of the STD NMR analysis depicting the interaction between LOX-1 and 5-LOX are depicted in Figure 9 and Figure 10. An examination of the differences in their spectra reveals that the peaks corresponding to both the aromatic and aliphatic protons of each compound exhibit decreased intensity compared to the reference ^1^H-NMR spectrum. This observation provides further confirmation of the interaction between the ligands and LOX-1 and 5-LOX. Furthermore, when examining the aromatic regions, the comparison between the ^1^H NMR spectrum of the complex formed by the compounds and soybean LOX-1 and the STD spectrum indicates that most of the aromatic protons engaged with the protein’s binding site. The reduced intensity of peaks in the STD spectrum, in comparison to the reference spectrum with no ligand, confirms the presence of a binding interaction. Also, the ligand’s signals appear less intense in the STD spectrum because the protein-bound ligand’s protons were saturated more effectively. This leads to a reduction in the intensity of those peaks in the STD spectrum. This signifies the strong binding of the ligand to the protein.

These findings align with our in silico results, showing that the aromatic rings of both molecules interact with 5-LOX. Furthermore, in **1**, most of the aromatic protons of both rings interacted with 5-LOX and the same occurred in **2**. On the other hand, in the LOX-1 enzyme, only the protons in **1** seemed to interact with the enzyme and only the protons in the aromatic ring (Figure 9, middle image). The Saturation Transfer Difference (STD) spectrum showed some detectable peaks. The limited solubility of the compounds in D_2_O may have prevented adequate interaction between the compounds and the solvent, thus hindering the observation of STD effects. The absence of some signals in the spectrum may be due to the low binding interactions between the ligand and the active site of a protein, as shown further in the in vitro results [15,16].

### 2.6. Absorption and Emission Spectra

**Figure 11:**
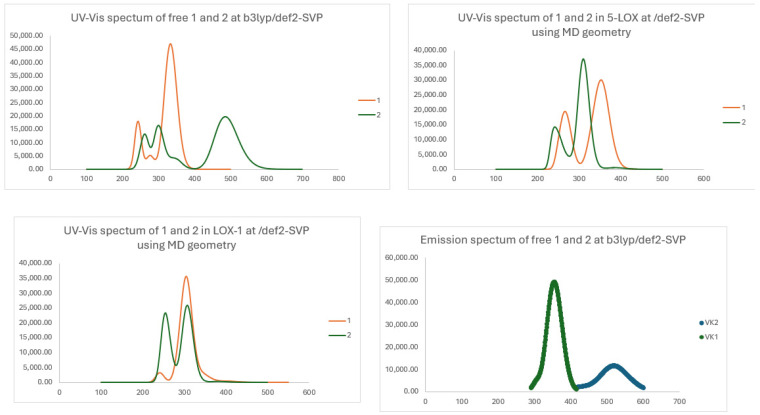
Absorption spectra of the free calculated compounds and those encapsulated in 5-LOX and in LOX-1, obtained using the B3LYP/def2-SVP method. Emission spectra of the free calculated compounds obtained using the B3LYP/def2-SVP method. The geometries of the encapsulated complexes’ structures, obtained via MD calculations.

Table 5 provides the λ values, energy difference, and oscillator strength values for the primary peaks observed in the visible–ultraviolet absorption spectra of the free chalcone molecules and encapsulated within each of the enzymes calculated via MD simulations. Figure 11 illustrates their absorption spectra. Table 6 provides the λ values, energy difference, and oscillator strength values of the UV–vis fluorescence spectra of the free chalcone molecules and encapsulated within each of the enzymes calculated via MD simulations.

The UV–vis absorption spectra of the hydroxy and methoxy chalcones show two absorption bands: the major one was observed at 340–390 nm while the minor one was found at 220–270 nm [17]. Recently, a series of chalcone derivatives were studied experimentally and theoretically and their UV–vis spectrum was measured. Among the studied chalcone derivatives, 1-(2-hydroxyphenyl)-3-(4-methoxyphenyl) prop-2-ene-1-one was found to have a methoxy group in position 4 [17]. Its main peak was measured at 366 nm and calculated at 375 nm, in good agreement with our calculated **1** compound, which has a methyl group in position 4 and a main absorption peak at 384 nm; see Table 5.

Regarding the absorption spectra of the present compounds, the first main UV–vis absorption peaks of **1** and **2** were observed at 384 and 485 nm, respectively, i.e., they differed by about 100 nm, showing that the substitution of the methyl group with a Cl atom, which is an electron donor, results in a significant blue shift in the main peak. This is interesting because the peak of **2** is in the blue region, which is not very common in chalcones. Note that the absorption excitation at 485 nm is associated with π-π* transitions involving conjugated systems present in the chalcone structure.

The UV–vis absorption spectra of the encapsulated compounds were calculated using their geometries, which were obtained via MD calculations of the molecular complexes of compounds @5-LOX and @LOX-1. For both compounds encapsulated by 5-LOX, their first absorbance band appeared at 386 nm, which is almost the same as that for the free **1**, but in the case of chalcone **2**, the encapsulation by 5-LOX resulted in a significant blue shift in the band. In LOX-1, the first UV–vis absorption band was observed at 403 nm for the encapsulated **1** and at 380 nm for the encapsulated **2**. The shifts in the first absorption bands were due to interactions between the chalcone derivatives and the 5-LOX and LOX-1. Overall, the corresponding energy differences in the absorption S_0_→S_1_ of the encapsulated chalcones ranged from 3.08 to 3.26 eV. Regarding the UV–vis emission spectrum (Figure 11), the first emission S_1_→S_0_ deexcitations of **1** and **2** were observed at 533 and 521 nm. However, for chalcone **1**, the oscillator strength was zero. Chalcone **1** presented with an intense emission peak at 337 nm. Organic compounds with fluorescence spectra in this area can be utilized as fluorescent labels and tags in biological imaging, diagnostics, and labeling applications [18,19]. These compounds can be conjugated to biomolecules or nanoparticles to selectively label specific cellular structures or biomarkers for visualization and detection purposes [20]. This computational study will be continued. Note that an understanding of the electronic structures of chalcones is necessary to provide chemical insights into the effect of substituents, since the absorption peaks are related to the electronic nature of the group of substituents. Computational studies on the effects of UV–vis absorption and emission spectra on the encapsulated chalcones’ derivatives on the active sites of enzymes will be conducted and the results will be analyzed to find useful candidates for biological imaging, diagnostics, and labeling applications.

**Figure 12:**
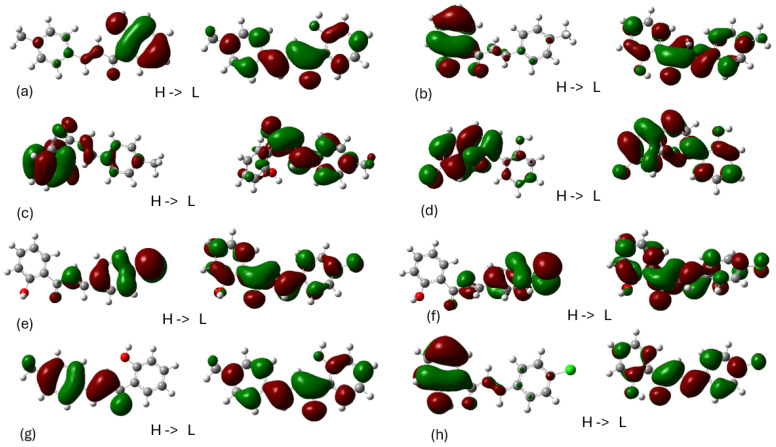
Frontier molecular orbitals (HOMO on the left and LUMO on the right) involved in the S_1_ absorption peaks in the (**a**) free 1, (**b**) 1 complexed with 5-LOX, (**c**) 1 complexed with LOX-1 after MD, (**d**) free 2, (**e**) 2 complexed with 5-LOX, and (**f**) 2 complexed with LOX-1, and frontier molecular orbitals involved in the S1→S0 fluorescence peaks in the (**g**) free 1 and (**h**) free 2.

The frontier molecular orbitals involved in the main UV–vis absorption peaks and fluorescence peaks are shown in Figure 12. Regarding the absorption spectra, the HOMO and LUMO orbitals of the ground state are localized to the aromatic rings. Specifically, for compound **1**, the HOMO orbital is localized to the one aromatic ring, while the LUMO orbital is localized to the aromatic ring next to methyl. Furthermore, in free chalcone **2**, the HOMO orbital is localized to the aromatic ring with chloride, while when it is complexed it is localized to the other aromatic ring. In all cases, the LUMO orbital is localized in the whole molecule. This phenomenon is a result of the molecular structure and the electronic environment surrounding the molecule. Regarding the emission spectrum, the HOMO orbital is localized in other ring, in contrast with the results obtained following absorption. The LUMO orbital is localized in the whole molecule, as the absorption spectrum. The frontier orbitals are essential to understand the molecule’s electronic structure, the nature of the conjugation, and the interaction of the molecule with light, which are fundamental to predict the reactivity, stability, and optical properties of organic compounds. Finally, the emission data allow us to understand the excited-state dynamics and relaxation pathways, which are key to designing molecules with specific light-emitting or light-absorbing characteristics.

### 2.7. MM/GBSA Calculations

MM/GBSA calculations were conducted for the most statistically significant ’protein–ligand’ complexes obtained via clustering the trajectories of the Desmond simulation. Based on the ∆G_bind values shown, compound **1** binds more favorably and specifically to the 5-LOX enzyme with a ∆G_bind value of −43.90 kcal/mol. From Table 7, it seems that in the 5-LOX enzyme, both compounds bind more strongly than the other enzymes. This is also in accordance with the docking scores.

### 2.8. In Vitro Evaluation of the Compounds Against Human 15-LOX-1 and Lipoxidase Enzymes

**Figure 13:**
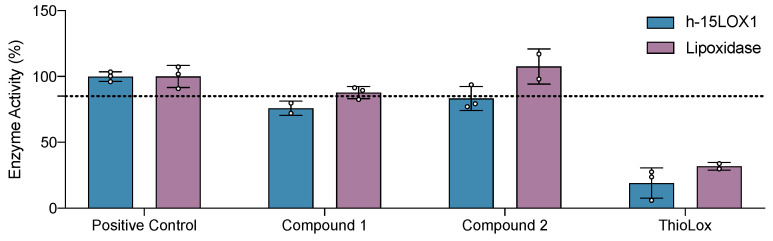
Compounds tested (at 100 μΜ) against human 15-LOX-1 and Lipoxidase enzymes. Positive control (absence of potential inhibitor) was set to 100% (dash line = 85%). The averages and standard deviations of the individual measurements are presented.

Finally, we investigated the inhibitory potency of the synthesized compounds against human 15-LOX-1 and Lipoxidase, which are isoenzymes of the LOX family. Our aim was to evaluate the selectivity profile of the compounds and identify any inhibition to the enzymes at μΜ concentrations (100 μΜ). As shown in Figure 13, the compounds exhibited minor inhibition minor inhibitions, indicating that they were bound to the proteins. However, none of the tested compounds proved to be very potent, as enzyme activity remained above 85% in both cases. ThioLox is a known inhibitor of human 15-LOX1 and was used as a control [21]. This is also in agreement with the STD results, in which no clear peaks in LOX-1 were observed.

The contradiction between the in silico predictions and weak enzymatic assay potency may occur due to several reasons. In silico models assess theoretical binding affinity but often overlook biological dynamics like solubility, stability, and the enzymes’ conformational flexibility. Experimental conditions, such as pH or poor compound orientation or steric hindrance in the active site, could reduce actual potency. Additionally, inaccuracies in the assay design or compound preparation may contribute to contradictions.

## 3. Materials and Methods

### 3.1. General Procedure for the Synthesis of Chalcones

2’-Hydroxy-chalcones **1** and **2** (Scheme 2) were synthesized via the Claisen–Schmidt condensation reaction between 2-hydroxy-acetophenoneand the appropriate benzaldehydes in basic conditions according to a previously published procedure [3].

More specifically, in an ethanolic solution of the 2-hydroxy-acetophenone (1 equiv) and a substituted benzaldehyde (1 equiv), 20% *w*/*v* aqueous KOH solution was added. The mixture was stirred at room temperature for 24 h and then the reaction mixture was cooled to 0 °C and acidified with a 10% aqueous HCl solution. A yellow precipitate was formed, which was filtered and washed with 10% aqueous HCl solution. The compounds were further purified via recrystallization.

The chemicals used for the synthesis of chalcones were purchased from Sigma-Aldrich (Burlington, MA, USA), Fluka (Buchs, Switzerland), Alfa-Aesar (Lancashire, UK) and Acros (Fukuoka, Japan) and were used without further purification. The reactions were monitored by TLC (Macherey-Nagel, Düren, Germany, 0.20 mm layer thickness plates).

2’-Hydroxy-4-methyl-chalcone (1) was prepared following the general procedure starting from 2-hydroxy-acetophenone (265 μL, 2.20 mmol) and p-methyl-benzaldehyde (259 μL mg, 2.20 mmol), dissolved in 5.4 mL ethanol and KOH (20% aqueous solution, 1.8 mL). The product was obtained after recrystallization from hexane/ethyl acetate as a yellow powder. Yield: 385.4 mg (70%). M.p. 117–119 °C (Ref. [3] 116–117).

^1^H NMR (400 MHz, CDCl_3_): δ (ppm) 12.90 (s,1H, OH), 7.95 (m, 2H, H6′&H_B_), 7.65 (d, 1H, J = 15.4 Hz, H_A_), 7.59 (d, 2H, J = 8.0 Hz, H2&H6), 7,52 (ddd, 1H, J = 8.6, 7.2, 1.6 Hz, H4′), 7.27 (d, 2H, J = 8.6 Hz, H3 & H5), 7.05 (dd, 1H, J = 8.4, 1.2 Hz, H3′), 6.97 (ddd, 1H, J = 7.87, 0.73 Hz, H5′), 2.42 (s, 3H,-CH_3_).

2’-Hydroxy-4-chloro-chalcone (2) was prepared following the general procedure, starting from 2-hydroxy-acetophenone (354 μL, 2.94 mmol) and p-chlorobenzaldehyde (413.2 mg, 2.94 mmol), dissolved in 7.2 mL ethanol and KOH (20% aqueous solution, 2.4 mL). The product was obtained after recrystallization from methanol as a yellow-orange solid. Yield: 620.4 mg (78%). M.p. 148–151 °C (Ref. [3] 153–156).

^1^H NMR (400 MHz, CDCl_3_): δ (ppm) 12.75 (s, 1H, OH), 7.91 (d, 1H, J = 8.0 Hz, H6′), 7.87 (d, 1H, J = 15.5 Hz, H_B_), 7.65–7.59 (m, 3H, H2, H6 & H_A_), 7,51 (t, 1H, J = 7.8 Hz, H4′), 7.42 (d, 2H, J = 8.1 Hz, H3 & H5), 7.04 (d, 1H, J = 8.4 Hz, H3′), 6.95 (t, 1H, J = 7.6 Hz, H5′).

### 3.2. Structure Assignment

The structure of the two molecules was identified using the 600 and 850 MHz spectrometers (Bruker Avance Spectrometer, Billerica, MA, USA) located at the National and Kapodistrian University of Athens, the National Institute of Chemistry in Ljubljana, and the University of Helsinki. This involved 1D and 2D homonuclear and heteronuclear experiments. The pulse sequences that were utilized were found in the spectrometer’s library. Finally, the spectra were processed and examined using the MestreNova (Santiago de Compostela, Spain) and TopSpin 4.2.0 software packages [22,23,24,25,26].

### 3.3. Saturation Transfer Difference (STD) NMR

For STD experiments, NMR samples were prepared by dissolving each chalcone in DMSO, followed by the addition of potassium phosphate buffer pH 7.2 in D_2_O, resulting in a total volume of 600 µL with a 20 mM buffer and pH 7.2 in 99.9% D2O. Specifically, chalcone **1** was dissolved in 192 µL and chalcone **2** in 206 µL of DMSO. Specifically, for the stock solutions of each chalcone, **1** was dissolved in 310 µL of DMSO and **2** in 250 µL of DMSO. The final concentration in the NMR tube (600 µL) was 1000 µM for the ligand and 1 µM for the enzyme, yielding a protein–ligand ratio of 1:1000. These samples were then subjected to STD experiments at 25 °C. 5-lipoxygenase and 1-lipoxygenase were chosen for the STD NMR experiments due to their biological relevance, their distinct functional roles, and the availability of stable preparations suitable for binding studies. These enzymes provided a clear framework to investigate specific ligand interactions within the lipoxygenase family [2,27,28,29].

STD NMR experiments were recorded on Bruker AV 850 MHz spectrometer (Bruker Biospin, Rheinstetten, Germany) using the Topspin 2.1 suite. The spectral width was 13,586.957 Hz. Pulse sequences provided by the Bruker libraries of pulse programs were used. Selective on-resonance irradiation frequency was set to 4.7 parts per million (ppm) with a saturation time of 2 s. The duration of the presaturation of 2 s was adjusted using n¼ 16 cycles. An off-resonance irradiation frequency for the reference spectrum was applied at 12.68 ppm.

### 3.4. Conformational Analysis

A conformational analysis was carried out using density functional theory (DFT) [30] at the BP86/def2-SVP level of theory [31,32]. This approach, known for its accuracy, involved optimizing all conformers and computing their frequencies to confirm their stability as true minima. For the extraction of results, various conformations from each molecule were selected to identify the conformation with the lowest energy value following systematic exploration. Specifically, conformers regarding the rotation of the -OH, -Ph, and -CH3 groups, as well as the trans–cis isomers, were studied. The lowest conformer was determined and its UV–vis absorption and emission spectra were calculated, as presented below. Finally, the theoretical data were compared with the experimental results obtained from the NMR spectra. All the calculations were conducted using ORCA software 4.0.1 in a vacuum [33].

### 3.5. Induced Fit Docking

Induced fit docking was utilized to explore the potential binding interactions between and the active sites of the LOX-1, 15-LOX, and 5-LOX enzymes. These enzymes were chosen as a potential target due to the findings of our literature review, which suggested that chalcones’ substructures could act as potent enzyme inhibitors [14,34]. Additionally, analysis with the Swiss Target webtool identified LOX as a promising target for the two molecules. The crystal structures used for the computational studies were PDB ID 5T5V [35], 2P0M [36], and 3O8Y [37]. In silico calculations were carried out using the Protein Preparation Wizard in the Schrödinger Suite to process the crystal structure. To address computational challenges related to the use of LOX as a metalloprotein, the presence of the Fe^3+^ cation in its active site was managed using the “create zero-order bonds to metals” tool in Schrödinger’s Maestro platform. This tool breaks pre-existing bonds with metals and forms new zero-order bonds between the metals and nearby atoms, while adjusting formal charges to align with the coordination geometry obtained from the X-ray data. The two compounds were then sketched in Schrödinger’s Maestro 2017-1 molecular modeling platform and minimized using Macromodel (Version 10) [38] and DFT calculations, as implemented in Schrödinger’s Maestro. The conformer analysis ensured the reliable identification of low-energy geometries using DFT, while molecular mechanics provided further minimization in the active site of the enzymes, combining accuracy and computational efficiency. LigPrep was employed for 3D model preparation, considering stereochemistry features, and the “add metal binding states” option was chosen to ensure successful binding. The optimization of geometries with the MacroModel retained their proper chiralities. The OPLS2005 [39] force field was used for minimization and protonation states were considered at a physiological pH. Chemically accurate 3D models were created using the Hammett and Taft methods combined with an ionization tool. The ligand structures were then minimized with water as the solvent and OPLS2005 as the chosen force field. To explore energetically favorable conformations, a mixed-torsional/low-sampling conformational search was conducted, with the most stable conformation selected for docking studies. The Induced Fit Docking (IFD) approach in the Schrödinger Suite was employed for these docking calculations. The ligand was docked using five energetically favorable conformations generated by Macromodel. For the center of the docking grid of 5T5V, the amino acids ILE839, HIS499, HIS504, HIS690, and ASN694 were used. For 3O8Y, the amino acids ASN407, HIS432, ILE673, HIS367, HIS372, and HIS550 were used while, for 2P0M, the amino acids ILE663, HIS545, ILE400, LEU597, ILE593, and VAL409 were used. Prior to docking, protein preparation involved constrained refinement, which included the automatic trimming of side chains based on B-factor and Prime refinement of the protein’s side chains. The Glide/XP docking tool was used, with the active site’s dielectric constant set to 80, and crystallographic waters were retained during the docking process.

### 3.6. Molecular Dynamics

The MD studies [40] were performed with SPC/E-modeled water molecules surrounding the drug–protein complex. The solution was neutralized using sodium (Na^+^) and chloride (Cl^−^) ions until the salt concentration reached 0.150 M NaCl. The N-terminal of the protein was modified with an acetyl group, while the C-terminal was left uncapped due to its role in the protein’s active site. Protein–ligand interactions were simulated with the OPLS2005 force field, and long-range electrostatic interactions were accounted for using the particle mesh Ewald (PME) technique [41,42] and a grid spacing of 0.8 Å. Van der Waals and short-range electrostatic interactions were smoothly truncated at 9.0 Å. Temperature was maintained using the Nose–Hoover thermostat [43], and pressure was controlled using the Martyna–Tobias–Klein [42] method. Periodic boundary conditions were applied, and the simulation box dimensions were (10.0 × 10.0 × 10.0) Å. The equations of motion were integrated using the multistep RESPA integrator [44] with an inner time step of 2 fs for bonded interactions and non-bonded interactions within a cutoff of 9 Å. An outer time step of 6.0 fs was used for non-bonded interactions beyond the cutoff. Each system underwent equilibration using the default protocol provided by Desmond [45]. The system was initially relaxed using Brownian dynamics simulations within the NVT ensemble at 310 K, applying restraints on the heavy atoms of the solute. This was followed by a relaxation step in the NPT ensemble without restraints for 1.0 ns, after which the production phase of the molecular dynamics (MD) simulation was initiated, lasting 200 ns to ensure there were sufficient data for analyzing the molecule’s binding to the protein cavity. The MD simulations were performed on workstations utilizing GPU-accelerated MD codes, with simulation performance evaluated through an examination of the RMSD convergence of the protein backbone’s Cα atoms and the ligand’s RMSD.

### 3.7. Theoretical Absorption and Fluorescence Spectra

UV–vis absorption and fluorescence spectra were computed using the TD-DFT method at the BP86/def2-SVP and B3LYP [46,47]/def2-SVP level of theory. Specifically, for the UV–vis absorption spectra, 10 singlet excited states were calculated for the free molecules optimized at the DFT level and encapsulated at LOX-5 and 1-LOX using the MD geometry. For the calculation of the emission spectra, the molecules were geometry-optimized for the first and second excited states. It was shown that the functional used, i.e., B3LYP, can provide accurate results for the absorption and emission spectra [48]. All calculations and the visualization of the results were carried out using Gaussian 16 [49] and ORCA.

### 3.8. Molecular Mechanics/Generalized Born Surface Area (MM/GBSA) Calculations

The stability of the protein–ligand complexes was evaluated using the MM/GBSA method, which calculates the free binding energy [50]. This approach was implemented for complex structures using the Prime module in Maestro software version 2024-4. MM/GBSA calculations were performed on the three most statistically significant ligand–protein complexes, identified through a cluster analysis of an MD trajectory. The VSGB solvation model, known for its realistic solvation parameterization, and the OPLS-2005 force field were employed to incorporate protein flexibility. The binding energy was computed using the following formula:

∆G_bind = E_complex (minimized) − E_ligand (minimized) − E_receptor (minimized).

### 3.9. Enzyme Inhibition Studies

Activity assay: The h-15LOX1 was expressed in BL21 (DE3) *E. coli* cells and the cell lysate was used for the activity assay, as described before [21,51,52]. Briefly, the conversion of linoleic acid to 13S-hydroperoxy-9Z,11E-octadecadienoic acid (13(S)-HpODE) was observed through UV absorbance at 234 nm over time with a ThermoFisher Varioskan Plate Reader and a Greiner Bio-One F-Bottom 96-well plate. The measurement took place for 20 min with an interval time of 20 sec. Only the linear part was used for the determination of the enzymatic activity, typically extending over the first 1–5 min depending on the enzyme concentration. After that, the conversion rate slowed down due to the consumption of the substrate. The activity assay was used to determine the optimum concentration of the cell lysate (×200 times dilution in assay buffer: 50 mM HEPES, 50 mM NaCl, pH 7.5). Linoleic acid (Sigma Aldrich, L1376) was diluted in ethanol. The same experimental approach was used for the determination of the optimal concentration of soybean Lipoxidase (Sigma Aldrich, Burlington, MA, USA L7395) (x16,000 times dilution in an assay buffer: 50 mM Tris, 50 mM NaCl, pH 7.5). 

Screening UV assay: For the evaluation of the inhibitory potency of the compounds the same experimental approach, based on the absorption of 13(S)-HpODE at 234 nm, was used. All the compounds were dissolved in DMSO at a final concentration of 4 mM. Then, they were diluted with the assay buffer and tested at 100 μΜ. Each compound was mixed with the diluted cell lysate/soybean Lipoxidase and, after a 10-minute incubation at RT, the linoleic acid was added at a final concentration of 25 μΜ. All the values were normalized by setting the absence of the inhibitor as 100%. Compounds in test samples with an enzyme activity of less than 50% were considered hits. Each measurement was performed in triplicate and all data were processed with Microsoft Excel Professional Plus 2021 and GraphPad Prism 9.0.0 software.

## 4. Conclusions

This research paper focuses on the elucidation and analysis of the structure of two bioactive compounds through a combination of NMR spectroscopy and computational analysis. The way these compounds interact at the smallest scales was discovered using a special experimental technique called STD NMR (Saturation Transfer Difference Nuclear Magnetic Resonance). This technique allows scientists to see how the compounds bind to other molecules. The results of these experiments are consistent with the compound structures that were predicted using computer simulations.

In silico experiments were conducted using 5-LOX, 15-LOX, and LOX-1. The results suggest a strong binding affinity of the compounds to both enzymes of 5-LOX and LOX-1 and, from the molecular dynamics, it seems that all the molecules remain in the active center of both enzymes. According to the SwissADME platform, all the derivatives exhibit no hepatotoxicity and comply with Lipinski’s Rule of Five. These findings suggest that they are safe and bioactive compounds, with potential for use in various biological and pharmacological targets. This research provides valuable insights for synthetic chemists interested in developing new structures for specific targets. Ultimately, the aim is to utilize in silico molecular modeling screening to synthesize molecules that selectively target certain biological entities.

## Figures and Tables

**Table 1:** Assignment of the experimental ^1^H NMR spectra of **1** and **2** in DMSO-d_6_.

Hydrogen 1	Chemical Shift (ppm)	Hydrogen 1	Chemical Shift (ppm)	Hydrogen 1	Chemical Shift (ppm)
CH_3_	2.36	HA	8.01	5’	6.96
2.6	7.82	HB	8.25	4’	7.57
3.5	7.29	3’	7.05	6’	8.27
OH	12.56				
**Hydrogen** **2**	**Chemical Shift (ppm)**	**Hydrogen** **2**	**Chemical Shift (ppm)**	**Hydrogen** **2**	**Chemical Shift (ppm)**
2.6	7.96	HB	7.83	4’	7.58
3.5	7.55	3’	7.01	6’	8.25
H_A_	8.05	5’	7.01		
OH	12.43				

**Table 2:** Table of signals observed in 2D-NOESY, along with their measured distances.

Observed Signal in 2D-NOESY Spectrum	Distance in Å
1	
H2-HB	2.192
2	
H2-HB	2.363
H6’-HA	1.882

**Table 3:** Docking results between the molecules and macromolecules in kcal/mol.

Compound	IFD Score (5-LOX)	IFD Score (LOX-1)	IFD Score (15-LOX)
**1**	−10.778	−9.657	−7.581
**2**	−11.630	−10.256	−7.634

**Table 4:** Interactions of the molecules with the enzymes.

Compound	Enzyme	Hydrogen Bonds	π-π Stacking
**1**	5-LOX	ASN425	HIS367, HIS372
	LOX-1		HIS504, TRP500
	15-LOX		HIS361, HIS366
**2**	5-LOX		HIS372, PHE421
	LOX-1		TRP500
	15-LOX		HIS361

**Table 5:** The λ (nm), energy difference ΔE (eV), and f-values of the main peak excitations and the selected UV–vis absorption peak excitations of the free compound and those encapsulated within LOX-5 and 1-LOX, obtained using the B3LYP/def2-SVP method.

	State	λ	ΔΕ	f
**1**	S_1_	383.5	3.755	0.3485
**1**_LOX-1	S_1_	403.1	3.076	0.0059
**1**_LOX-1	S_3_	306.3	4.048	0.4806
**1**_5-LOX	S_1_	385.6	3.215	0.0005
**1**-OCH_3_^a^	Expt ^a^	366 nm		
	S_1_^a,b^	375		
**2**	S_1_	485.3	2.555	0.2922
**2**_LOX-1	S_1_	380.8	3.256	0.0043
**2**_LOX-1	S_3_	305.2	4.062	0.2364
**2**_5-LOX	S_1_	386.1	3.211	0.0081

^a^ Reference 17; 1-(2-hydroxyphenyl)-3-(4-methoxyphenyl) prop-2-ene-1-one. Methoxy group instead of methyl group in position 4. ^b^ B3LYP/6-311++G(d,p).

**Table 6:** The λ (nm), energy differences ΔE (eV), and f-values of the S_1_→S_0_ de-excitations of the emission spectra of the free compounds obtained using the B3LYP/def2-SVP method.

	λ	ΔΕ	f
**1**	532.5	2.328	0.0000
^a^	431.9	2.871	0.0030
	337.2	3.677	0.5410
**2**	521.0	2.379	0.1712
^a^	459.4	2.699	0.0299

^a^ S_3_→S_0_ de excitations.

**Table 7:** MM/GBSA values.

Compound_Enzyme	∆G_Bind Value (kcal/mol)
**1**_15-LOX	−35.90
**2**_15-LOX	−27.32
**1**_5-LOX	−43.90
**2**_5-LOX	−31.07
**1**-LOX-1	−34.49
**2**-LOX-1	−23.80

## Data Availability

The data presented in this paper are available in the Supporting Information.

## Supplementary Materials

The following supporting information can be downloaded at: https://www.mdpi.com/article/10.3390/ph18010088/s1, Figure S1: 2D-COSY NMR spectrum of compound **1**; Figure S2: 2D-NOESY NMR spectrum of compound **1**; Figure S3: 13C NMR spectrum of compound **1**; Figure S4: 2D-HSQC NMR spectrum of compound **1**; Figure S5: 2D-HMBC NMR spectrum of compound **1**; Figure S6: 2D-NOESY NMR spectrum of compound **2**; Figure S7: 13C NMR spectrum of compound **2**; Figure S8: 2D-HSQC NMR spectrum of compound **2**; Figure S9: The spectra were recorded in DMSO-d6 using a Bruker AC 400 MHz spectrometer at 25 °C. Figure S10: A timeline representation of protein–ligand contacts for compounds **1** (a) and **2** (b) during simulation with 5-LOX; Figure S11: A timeline representation of protein–ligand contacts for compounds **1** (a) and **2** (b) during simulation with LOX-1; Figure S12: A timeline representation of protein–ligand contacts for compounds **1** (a) and **2** (b) during simulation with 15-LOX.
