# Supplementary material for: Synthesis of Anti-Inflammatory Drugs’ Chalcone Derivatives and a Study of Their Conformational Properties Through a Combination of Nuclear Magnetic Resonance Spectroscopy and Molecular Modeling
Source: Pharmaceuticals (Basel). 2025 Jan 13;18(1):88. doi: 10.3390/ph18010088 (PMC11768734; doi:10.3390/ph18010088)
Supplement: Supplementary file 1 [file pharmaceuticals-18-00088-s001.zip › pharmaceuticals-3396157-supplementary.pdf]

SUPPORTING INFORMATION

Synthesis of anti-inflammatory drugs' chalcone derivatives and a study of their conformational properties through a combination of NMR spectroscopy and molecular modeling

Nikitas Georgiou[1], Andromachi Tzani[2], Kyriaki Vavougyiou[1], Christos Papadopoulos[3], Nikolaos Eleftheriadis[3], Primoz Sket[4], Demeter Tzeli[5,6], Tuomas Niemi-Aro[7], Anastasia Detsi[2], Thomas Mavromoustakos[1*]

[1]Laboratory of Organic Chemistry, Department of Chemistry, National and Kapodistrian University of Athens, Panepistimioupolis Zografou, 11571 Athens, Greece

[2]Laboratory of Organic Chemistry, Department of Chemical Sciences, School of Chemical Engineering, National Technical University of Athens, Heroon Polytechniou 9, Zografou Campus, 15780 Athens, Greece

[3]Department of Chemistry, University of Crete, Voutes, 70013 Heraklion, Greece

[4]Slovenian NMR Centre, National Institute of Chemistry, SI-1001 Ljubljana, Slovenia

[5]Laboratory of Physical Chemistry, Department of Chemistry, National and Kapodistrian University of Athens, Panepistimioupolis Zografou, 11571 Athens, Greece

[6]Theoretical and Physical Chemistry Institute, National Hellenic Research Foundation, 48 Vassileos Constantinou Ave., 11635 Athens, Greece

[7]Institute of Biotechnology, Helsinki Institute of Life Sciences, P.O 65 (Viikinkaari 1), 00014 University of Helsinki

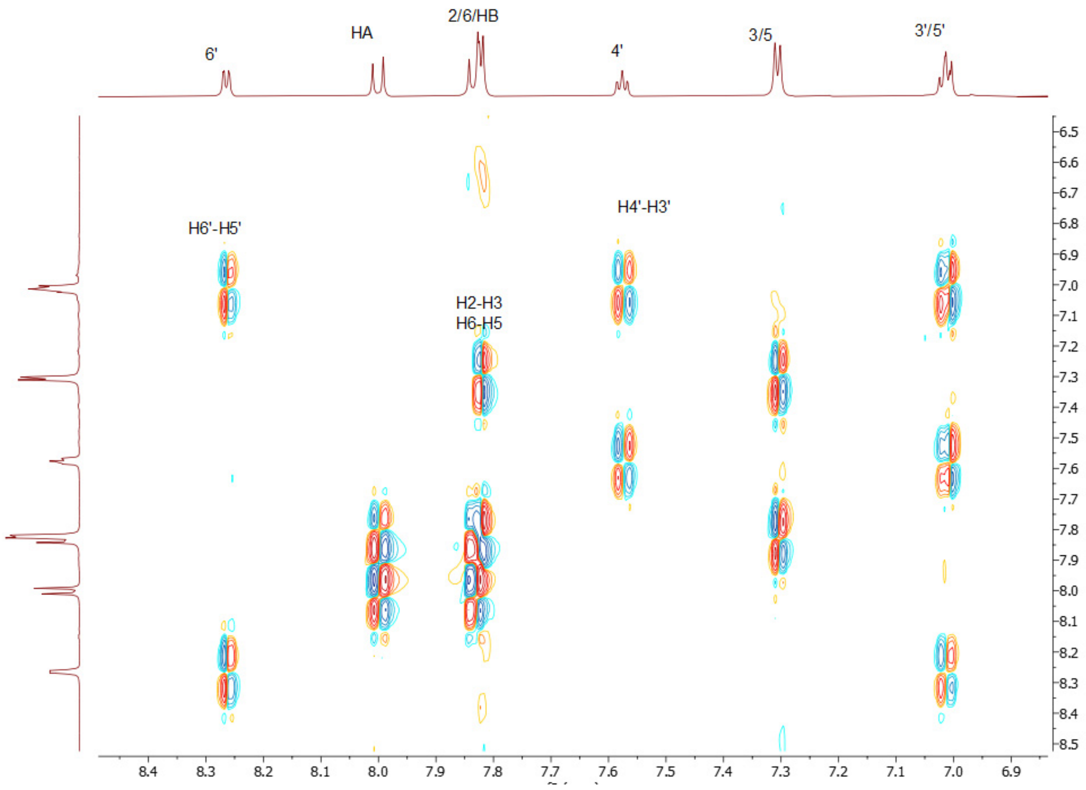

Figure S1: 2D-COSY NMR spectrum of compound 1. The spectra were recorded in DMSO-d_6 using a Bruker AC 850 MHz spectrometer at 25 °C.

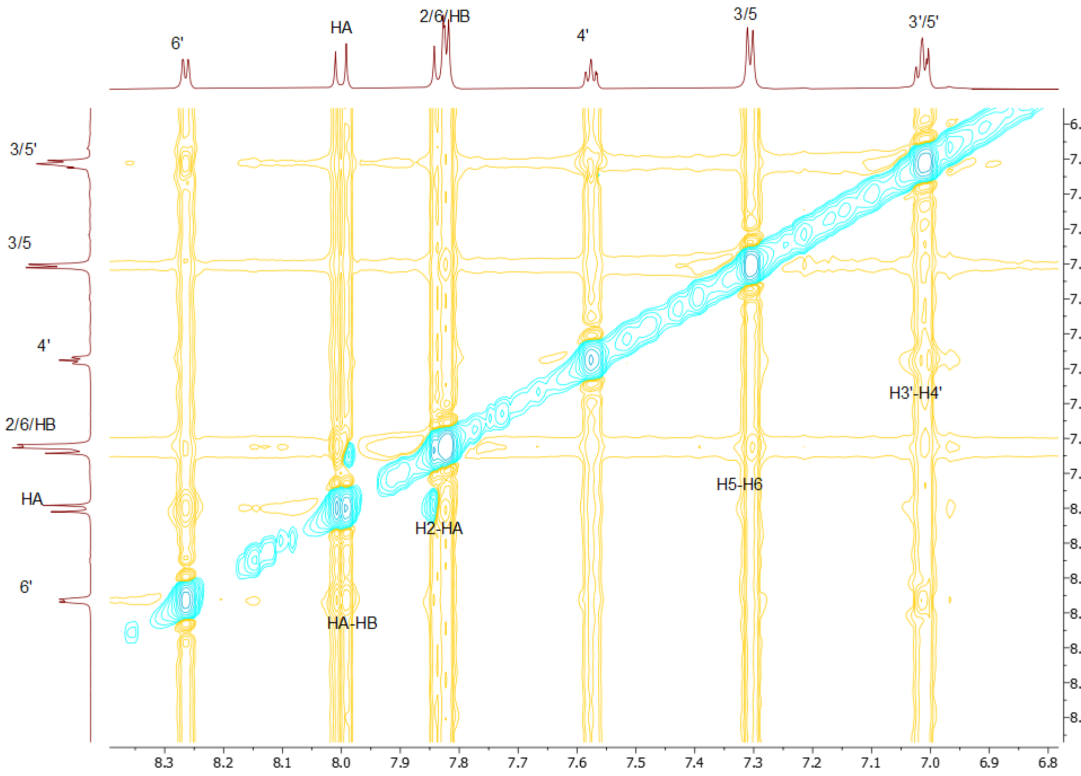

Figure S2: 2D-NOESY NMR spectrum of compound 1. The spectra were recorded in DMSO-d_6 using a Bruker AC 850 MHz spectrometer at 25 °C.

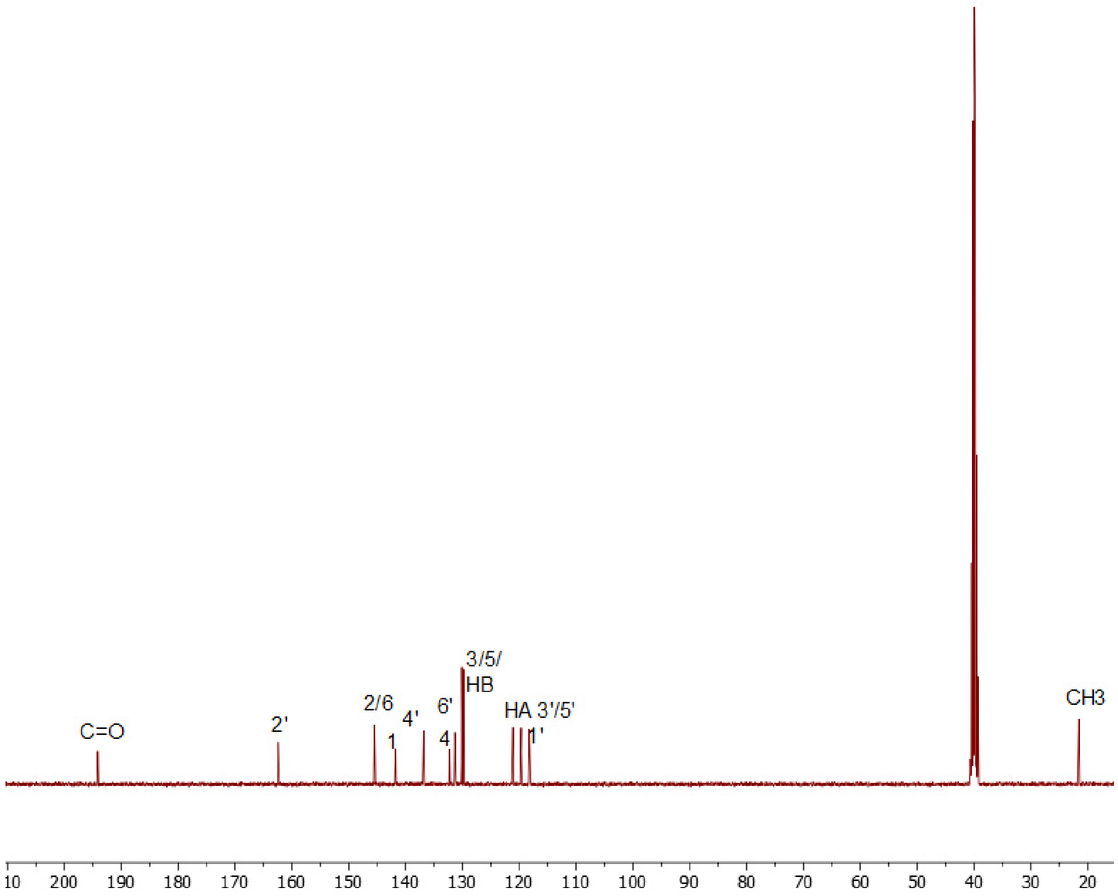

Figure S3: ^{13}C NMR spectrum of compound 1. The spectra were recorded in DMSO-d_6 using a Bruker AC 850 MHz spectrometer at 25 °C.

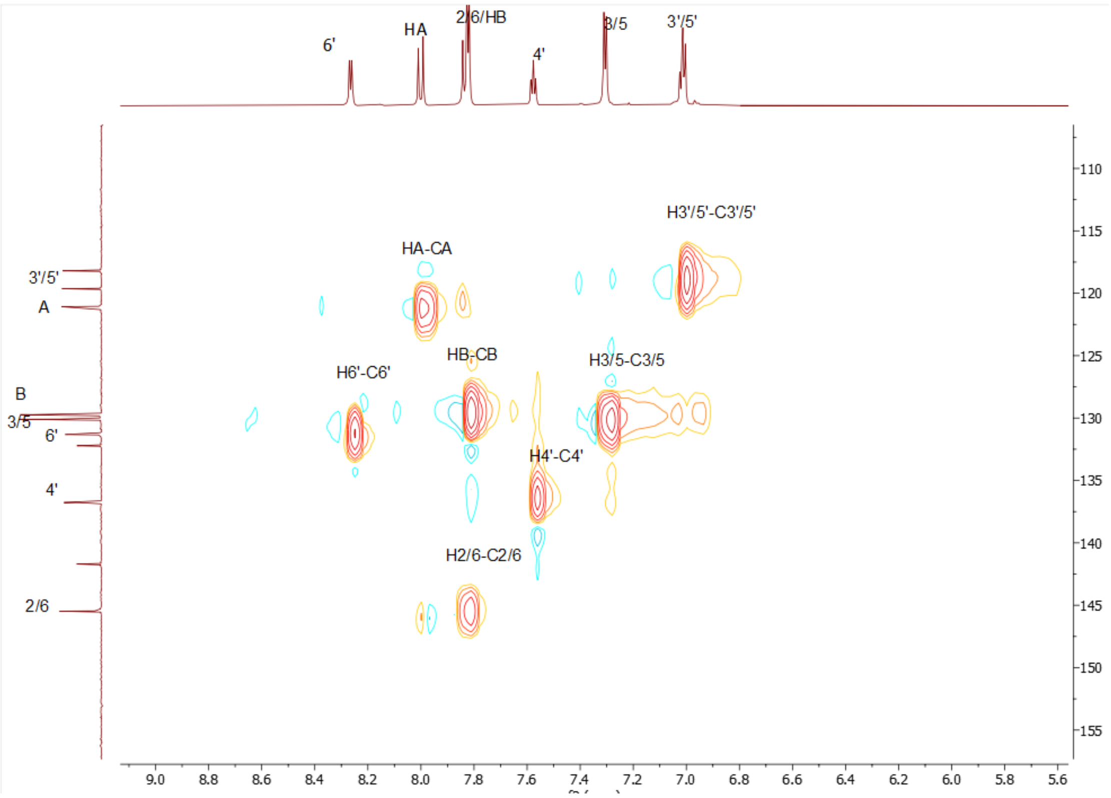

Figure S4: 2D-HSQC NMR spectrum of compound 1. The spectra were recorded in DMSO-d_6 using a Bruker AC 850 MHz spectrometer at 25 °C.

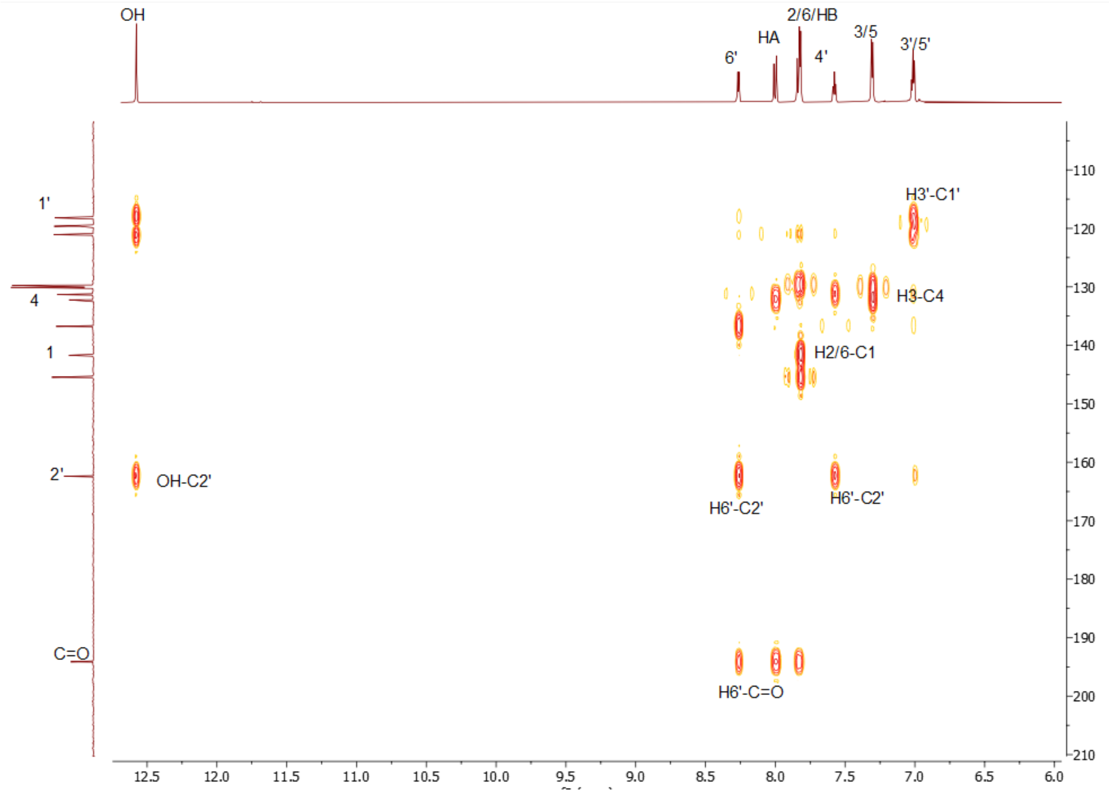

Figure S5: 2D-HMBC NMR spectrum of compound 1. The spectra were recorded in DMSO-d_6 using a Bruker AC 850 MHz spectrometer at 25 °C.

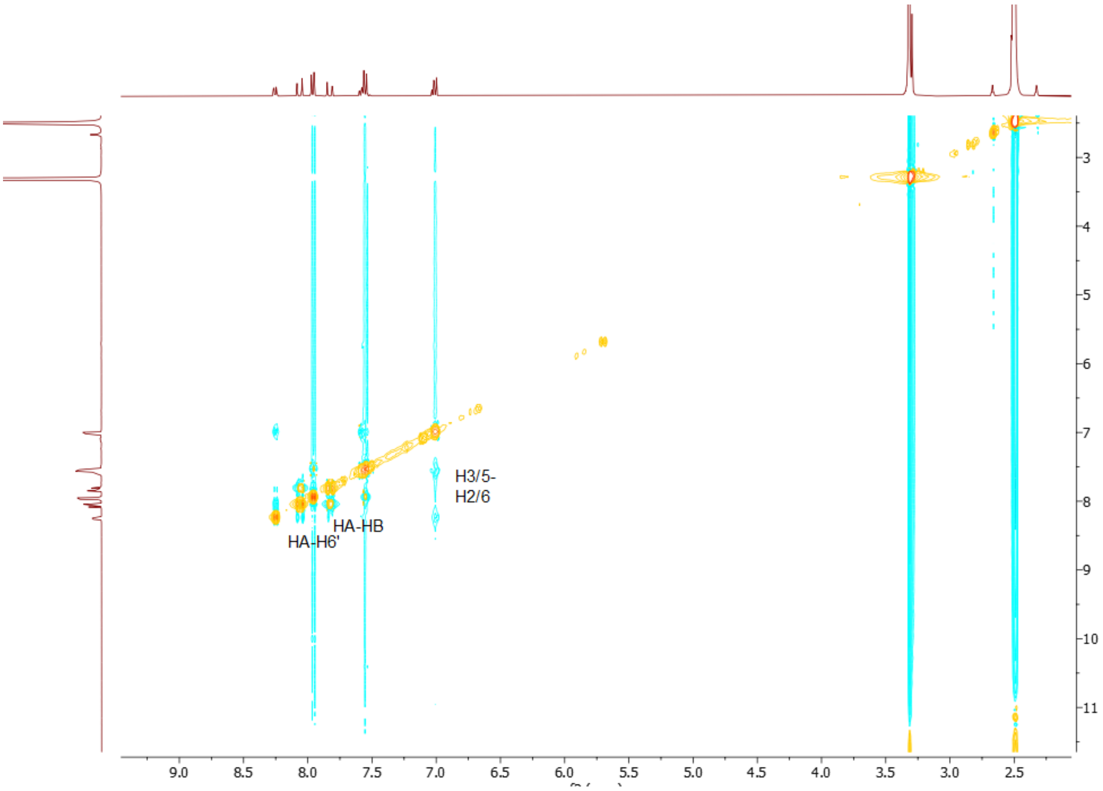

Figure S6: 2D-NOESY NMR spectrum of compound 2. The spectra were recorded in DMSO-d_6 using a Bruker AC 400 MHz spectrometer at 25 °C.

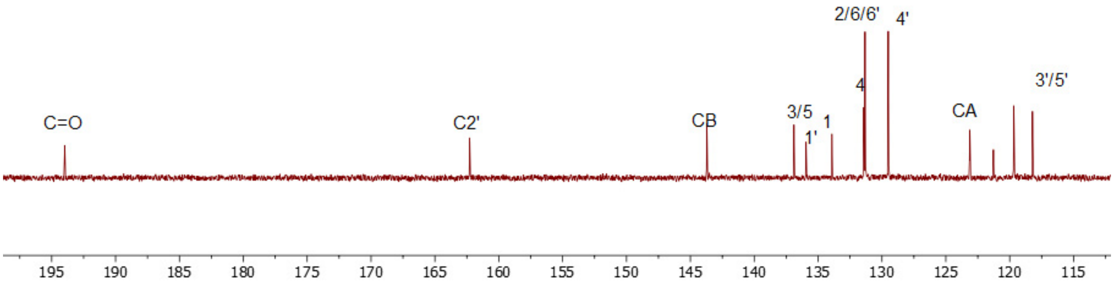

Figure S7: ^{13}C NMR spectrum of compound 2. The spectra were recorded in DMSO-d_6 using a Bruker AC 400 MHz spectrometer at 25 °C.

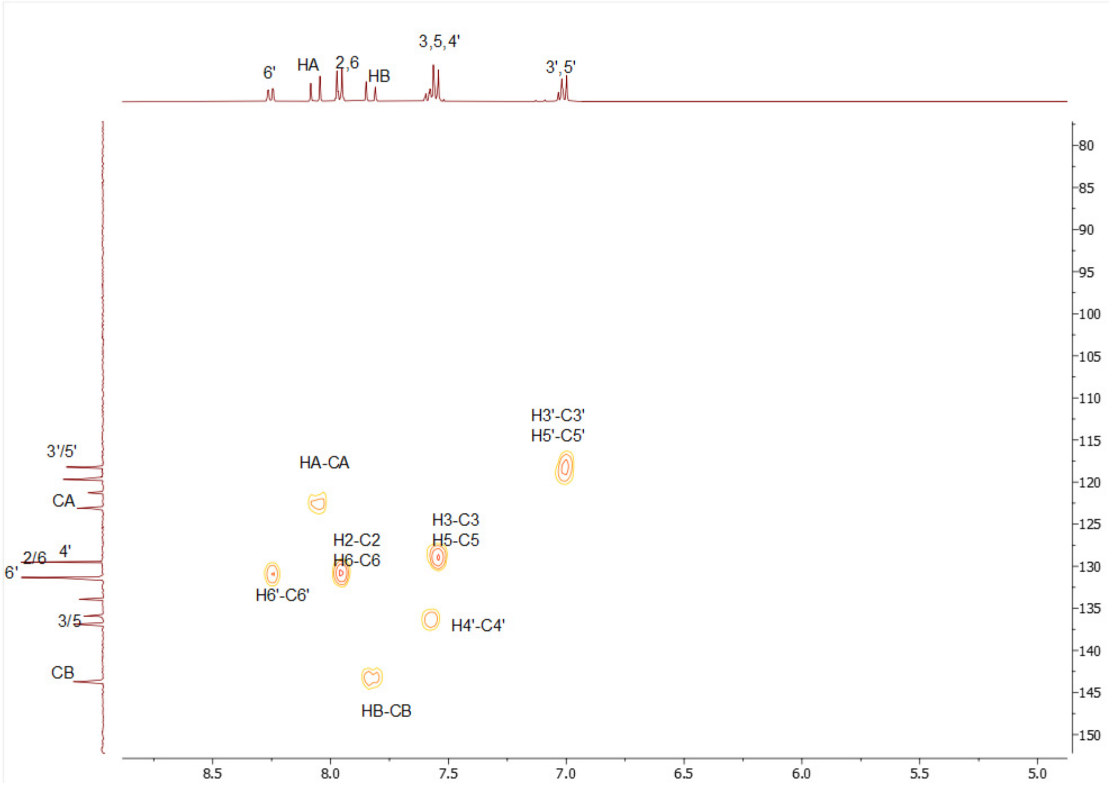

Figure S8: 2D-HSQC NMR spectrum of compound 2. The spectra were recorded in DMSO-d_6 using a Bruker AC 400 MHz spectrometer at 25 °C.

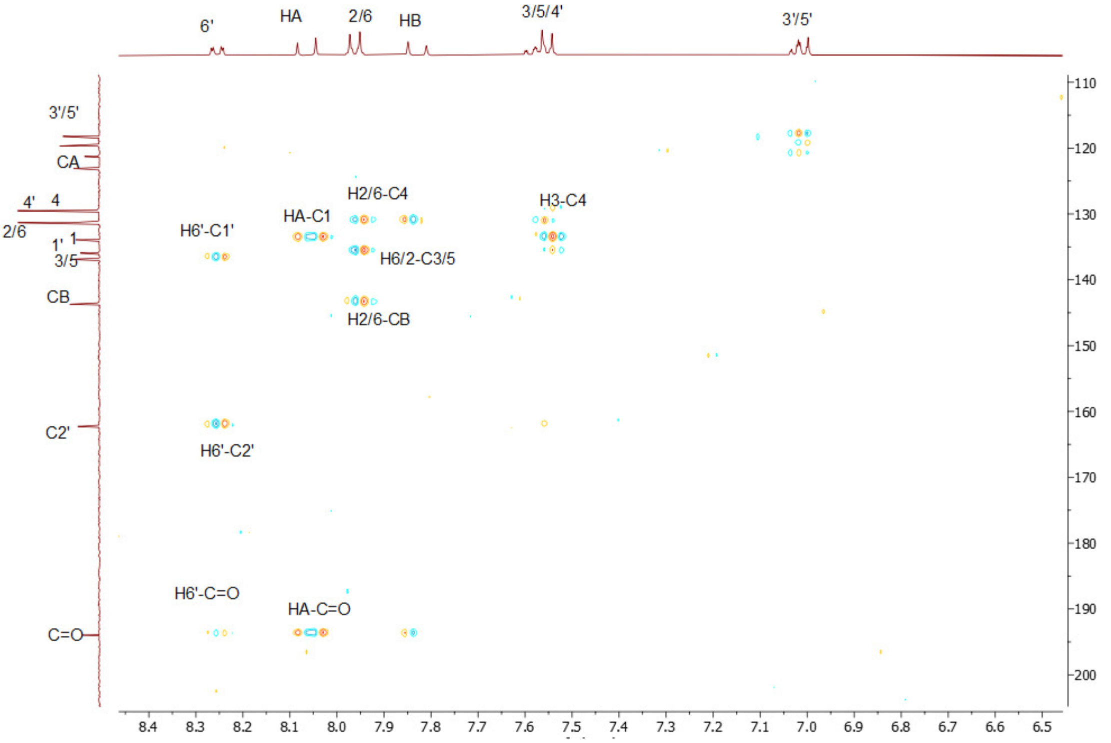

Figure S9: 2D-HMBC NMR spectrum of compound 2. The spectra were recorded in DMSO-d_6 using a Bruker AC 400 MHz spectrometer at 25 °C.

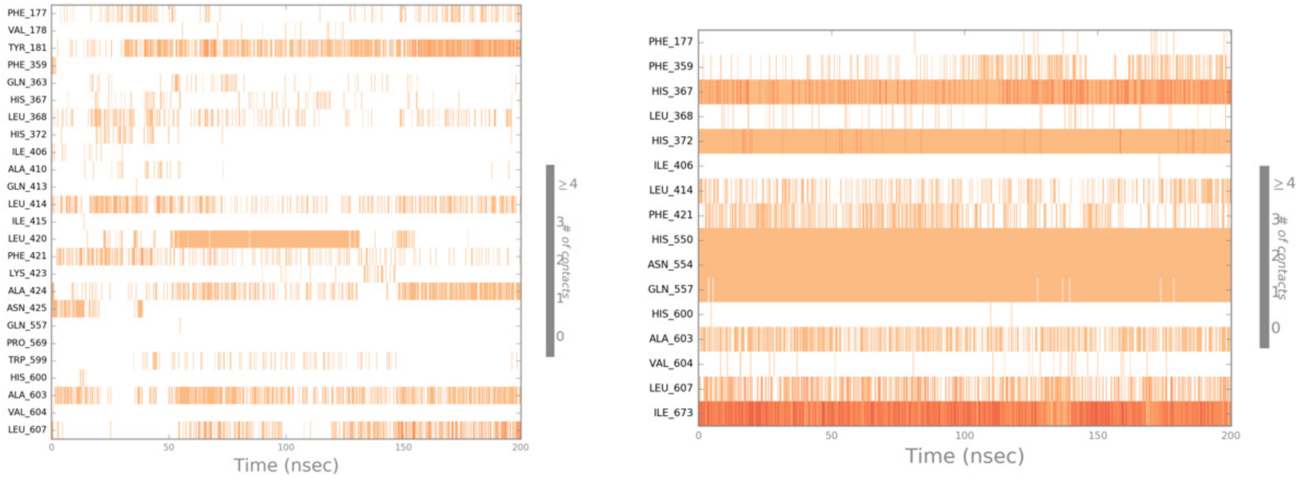

(a) (b)

Figure S10: A timeline representation of protein–ligand contacts for compounds **1** (a) and **2** (b) during simulation with 5-LOX.

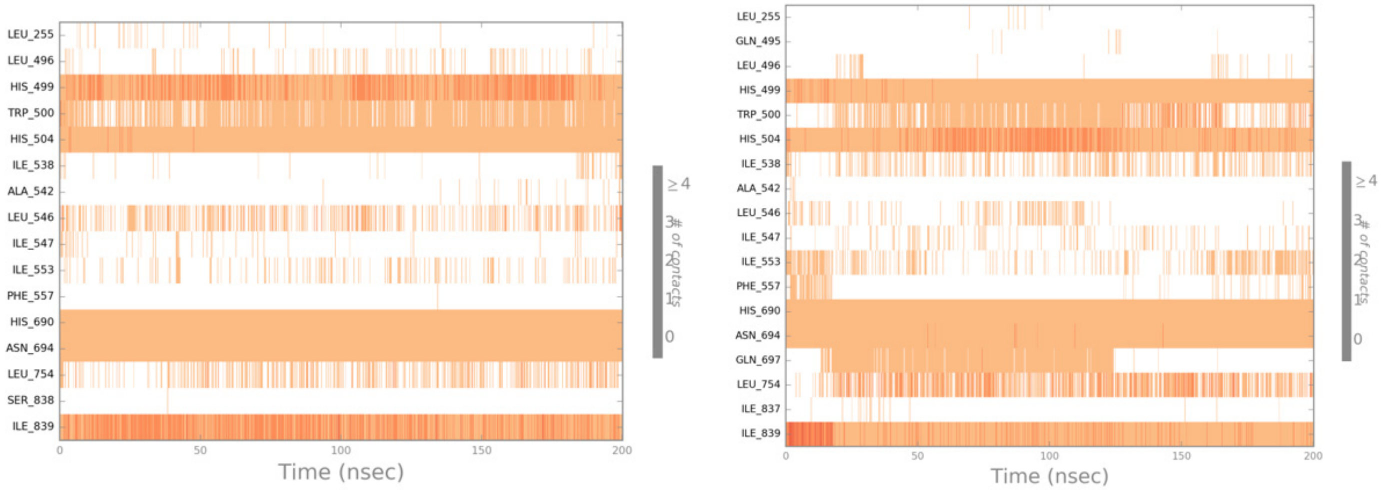

(a) (b)

Figure S11: A timeline representation of protein–ligand contacts for compounds **1** (a) and **2** (b) during simulation with LOX-1.

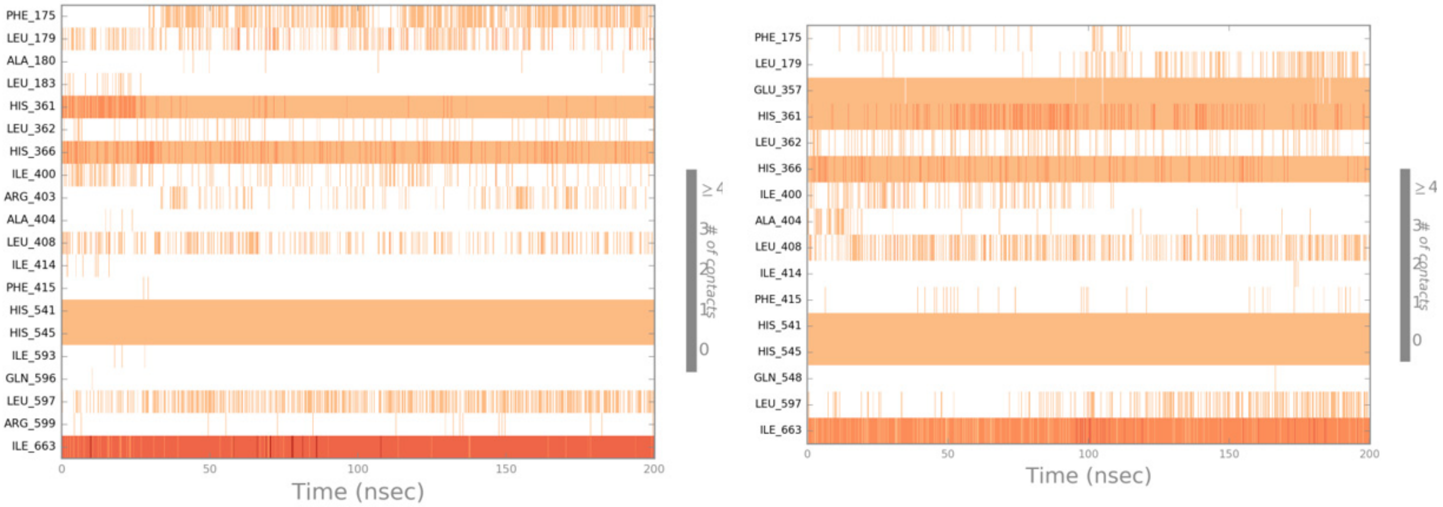

(a) (b)

Figure S12: A timeline representation of protein–ligand contacts for compounds **1** (a) and **2** (b) during simulation with 15-LOX.
